# Cloning, Transformation and Expression of Proinsulin Gene in Tomato (*Lycopersicum esculentum* Mill.)

**DOI:** 10.17795/jjnpp-7779

**Published:** 2014-02-20

**Authors:** Behnoush Soltanmohammadi, Mokhtar Jalali-Javaran, Hamid Rajabi-Memari, Mehdi Mohebodini

**Affiliations:** 1 Department of Biotechnology and Plant Breeding, Faculty of Agriculture, Tarbiat Modares University, Tehran, IR Iran; 2 Department of Agronomy and Plant Breeding, Shahid Chamran University, Ahvaz, IR Iran; 3 Department of Horticulture Science, University of Mohaghegh Ardabili, Ardabil, IR Iran

**Keywords:** Diabetes Mellitus, Insulin, Gene Transfer Techniques, *Lycopersicum esculentum*, *Agrobacterium tumefaciens*

## Abstract

**Background:**

Plants are among promising and suitable platform systems for production of recombinant biopharmaceutical proteins due to several features such as safety, no need for fermentation, inexpensive investment, and fast and easy scale-up. Human insulin is one of the most widely used medicines in the world. Up to now different expression systems including *Escherichia coli*, yeast and CHO have been exploited for producing recombinant human insulin and a variety of different recombinant insulin are extensively used.

**Objectives:**

This study reports on the transformation and expression of proinsulin gene in tomato plants for the first time in Iran.

**Materials and Methods:**

This study reports the cloning, transformation and expression of proinsulin gene in tomato plants. Specific primers were designed and used for PCR amplification and cloning of the proinsulin gene in the plant expression vector pCAMBIA1304. The recombinant construct was transferred into *Agrobacterium tumefaciens* strain LBA4404, and used for *Agrobacterium* mediated stable transformation of tomato plants. Presence of the desired gene in transgenic lines was confirmed through colony PCR and sequencing. The expression of the protein in transgenic lines was confirmed by immunodot blot assay.

**Results:**

The presence of the proinsulin gene in the genomic DNA of transgenic tomato was confirmed by PCR. Also total protein of transgenic tomato was extracted and the expression of proinsulin was detected using dotblot assay.

**Conclusions:**

This survey addresses the possibility of proinsulin gene transfer and expression in tomato transgenic lines. This study can be used as a basis for future researches to produce human proinsulin in tomato and other candidate plants.

## 1. Background

About 0.7% of the world population is living with insulin-dependent diabetes with an increasing number of patients. In industrial countries, type 1 diabetes is the third most prevalent condition contributing to mortality after cardio-vascular disease and cancer (1). Therefore, the requirement for recombinant insulin is increasing nowadays (2). Various methods for production of proinsulin in the cytoplasm of *Escherichia coli* as insoluble inclusion bodies have been demonstrated and are commercially used. The major advantage of these approaches is that proinsulin can be produced in large scale, but the intricate process of refinement and the formation of accurate disulfide bonds during folding are critical cost factors (2). Other production systems using other microbial fermentation, insect and mammalian cell cultures, and transgenic animals have disadvantages regarding cost, scalability, product safety and accuracy. So, a simple and low cost system allowing large-scale production of safe recombinant proteins is highly demanded (3). Insulin is a polypeptide with 51 amino acids and has two chains of A and B. A chain has 21 amino acids and has an internal molecular disulphide bond. B chain has 30 amino acids and binds to the A chain with two disulphide bonds. Insulin is produced in pancreatic beta-cells as preproinsulin and has signal peptides which conduct it to the endoplasmic reticulum. Proinsulin is produced by cleavage of the first 24 amino acids from amino terminus of preproinsulin (4). Proinsulin has a C peptide between the A and B chains, and separates in secreting vesicle (2). Studies have shown that proinsulin has a longer half-life than insulin in humans (5). The application of molecular biology and biotechnology in the 1990s displayed that many molecular medicines could be synthesized in plants (6).

Plants have been used for medicinal and industrial goals for many centuries, but it is recently possible to use them as a bio-platform for the expression of recombinant proteins. Plants are proper candidates as bio-factories because of several features, like ease of genetic engineering steps, no need for complicated steps of fermentation, very fast scale-up, high expression, and glycosylation ability (7). In 1986, transgenic tobacco and calluses of sunflower could produce growth hormone, which can be used in human treatment (8, 9). During the last decades, many different plant platforms have been used to produce recombinant proteins (10-13). Plants based medicines are considered as safe expression platforms for delivery of recombinant medicines for therapeutic use and easy retention and distribution. There is greater public acceptance for plants derived medicines compared to other platforms like Chinese hamster ovary (CHO), yeast, etc. making plants favorable hosts as expression platforms for providing a recombinant biopharmaceuticals (14-16). After production of transgenic tobacco and sunflower in 1986, progress was made in the molecular farming era. In 1989, the first plant antibody, an immunoglobulin G, was produced in tobacco plants (17). In 1990, Human Serum Albumin was expressed and secreted in the media of suspension cultures (18). In 1992, the first industrial enzyme, alpha amylase, was produced in tobacco (19). The first plantigen i.e. hepatitis B surface antigen (HBsAg) was expressed in tobacco during the same year (20). After these achievements the researchers intensified their surveys on posttranslational processes, high expression and commercialization. Avidin was the first commercialized protein which used Maize as a platform system in 1997 (21). The possibility of glycosylation of recombinant protein in plants was studied in 1999. The mouse IgG N-Glycosylation expressed in transgenic tobacco, was evaluated and proven. The glycan analysis revealed that the active glycoprotein similar to their native glycosylation happened in plant expression systems (22). Tomato (*Lycopersicum esculentum* Mill.) is one of the most important and favorable vegetable crops. It is an ideal candidate plant for the production and delivery of oral vaccines. Being a short-duration crop and having the ability to grow in greenhouses adds to its advantages for exploring the possibilities of using this crop for biopharmaceutical production (23). Tomato has served as a model plant for cloning agronomically main genes in dicotyledonous plants (24).

## 2. Objectives

In this study, we reported on the transformation and expression of proinsulin gene in tomato.

## 3. Materials and Methods

### 3.1. Bacteria and Vectors

**Figure 1.**
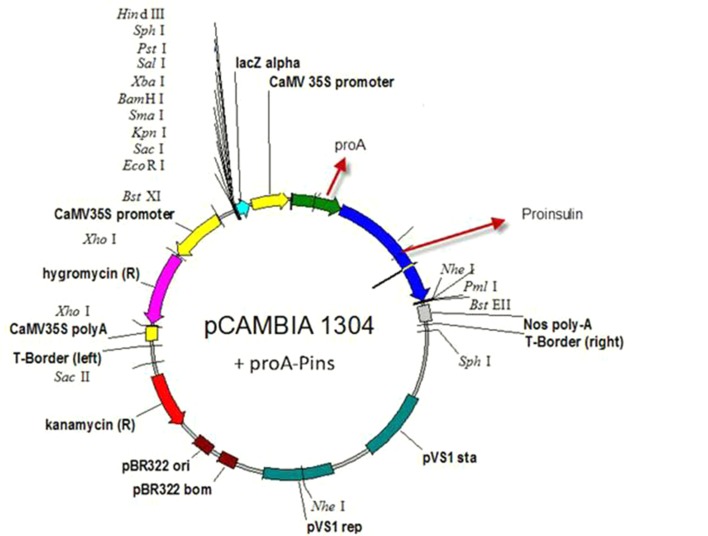
The Schematic Representation of Plant Expression Vector pCAMBIA1304 The human proinsulin gene and the Immunoglobulin G-binding protein A of *Staphylococcus aureus* (pro A-Pins) were cloned instead of the reporter genes.

*Agrobacterium tumefaciens* strain LBA4404 was used for gene transformation. The strain included the expression vector of pCAMBIA1304 (CAMBIA Co. Australia) harboring the human proinsulin gene. This expression vector having a resistance gene for kanamycin (NPT II) was used for selection of the transformed bacteria, and the hygromycin resistance gene was used to create hygromycin resistance in plants to screen the transgenic line. CaMV35S (Cauliflower Mosaic Virus promoter which induces high level of transcription) promoter, NOS (Nopaline Synthase terminator which induces termination process rate) terminator sequence, the reporter genes of GUS and GFP (as reporter genes) encoding beta-glucuronidase and green florescent protein was used in front of the 35S promoter, and the sequences for restriction enzymes of Bst EII and Nco I were used to replace the desired gene with the reporter genes (Figure 1). The human proinsulin gene and the Immunoglobulin G-binding protein A of *Staphylococcus aureus* (pro A-Pins) were cloned instead of the reporter genes. Proinsulin gene was obtained from the Pasteur Institute, Iran. The coding sequence is:

ATGGCGGGATTNAACCAATTTAATAAGGAACAACAAAATGCTTTCTATGAAATCTTACATTTACCTAACTTAAATGAAGAACAACGCAATGGTTTCATCCAGAGCTTAAAAGATGACCCAAGCCAAAGCGCTAACCTTTTAGCAGAAGCTAAAAAGCTAAATGATGCACAAGCACCAAAAGCTGATAACAAAGGATCCCGTCGCTTTGTTAACCAACACCTGTGCGGTTCTCACCTGGTTGAAGCTCTGTACCTGGTTTGCGGTGAACGTGGTTTCTTCTACACCCCGAAGACCCGTCGTGAAGCTGAAGACCTGCAGGTTGGTCAGGTTGAACTGGGTGGTGGTCCGGGTGCTGGTAGCCTGCAACCGCTGGCTCTGGAAGGTTCTCTGCAGAAGCGTGGTATCGTTGAACAGTGCTGCACCTCTATCTGCTCTCTGTACTACCAACTGGAAAACTACTGCAAC

**Figure 2.**
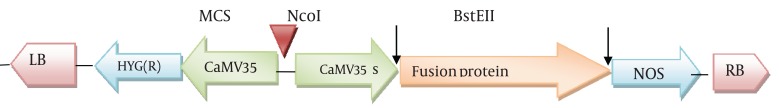
T-DNA Region of pCAMBIA-proinsulin LB and RB, Left and Right borders; HYG (R), hygromycin selectable marker; CaMV35s, cauliflower mosaic virus promoter; NcoI and BstEII, restriction sites and NOS, Nopaline.

Blue is *Staphylococcus aureus* spa gene for immunoglobulin G binding protein A", and black is proinsulin (without signal peptide) (signal peptide is ATGGC CCTGTGGATG CGCCTCCTGC CCCTGCTGGC GCTGCTGGCC CTCTGGGGAC CTGACCCAGC CGCAGCC), which has been deleted because there was no need for targeting recombinant protein (Preproinsulin is including signal peptide). Proinsulin is converted into the bioactive hormone insulin by removal of its connecting peptide (C-peptide). PROTEIN A can cause high expression of recombinant proinsulin gene and high stability of recombinant proinsulin protein produced in the *Escherichia coli*, and this protein causes easier purification of human proinsulin bound to it. Proinsulin is converted to bioactive hormone insulin by carboxypeptidase-H (CP-H) from Arg-Arg and Lys-Arg sites in secreting vesicle (25, 26). The sequence of fusion protein A was placed at the 5´end, and the sequence of human proinsulin was placed at the 3´ end of the template sequence (27) (Figure 2). The sequence of Protein A would be separated from proinsulin by endopeptidase activity from Arg-Arg site.

### 3.2. Primers

Primer design was performed by the Oligo 4 software, and two primers were used in this study. The forward primer was: 5′-CATGCCATGGAAGCGGGATTCAACCAATTTAATAAGG -3′, and the reverse primer was: 5′-CCGGTCACCTCATTAGTTGCAGTAGTTTTCCAG-3′.

### 3.3. PCR Amplification and Cloning

The PCR mixtures were denatured at 94˚C for 5 minutes followed by 35 cycles for 1 minute at 94˚C, 1 minute at 59˚C for annealing, 1 minute at 72˚C for extension, and finally incubated at 72˚C for 5 minutes. The thermal cycler was Quanta-biotech, auto Q server model. Expected PCR product size was about 500 bps. Amplified human proinsulin gene was extracted from agarose gel and digested with restriction enzymes, followed by insertion into pCAMBIA1304, which was previously digested with the same enzymes. The T-DNA of pCAMINS included a selectable marker gene construct for hyg B resistance, and a construct of ProA-Pins fusion sequence. DNA of the binary vector was transferred into the *Agrobacterium* by the freeze-thaw method.

### 3.4. Agrobacterium-mediated Transformation and Regeneration of Transgenic Tomato

In this study, three cultivars of tomato were used for transformation. Seeds of tomato (Cal J, Rio Grande, Urbana) were surface sterilized by 70% ethanol for 30 seconds, and then rinsed in distilled water for 1 minute. Then seed sterilization continued by immersing in 3% NaClO and rinsed three times with sterile distilled water. Sterile seeds were germinated in the dark at 25˚C on Murashige and Skoog (MS) (28) medium for seedling germination. After 7 days cotyledons were selected for transformation. The middle section of the cotyledons was used for transformation. These cotyledons were placed in pre-culture medium (PM) and incubated for 48 hours at 25˚C in a photoperiod of 16:8 (L:D) hours. Single colonies of the *A. tumefaciens* carrying the modified vector pCAMBIA1304 were grown overnight at 28˚C in LB medium with 50 mg/L kanamycin and 80 mg/L streptomycin. The culture was centrifuged at 3000 rpm for 10 minutes when it reached OD600 of 0.5 and the supernatant was discarded followed by addition of 20 mL MS liquid medium containing 200 µL acetosyringone. Then, explants were inoculated in infection medium (IM) for 30 minutes at room temperature. The explants were transferred into sterile paper to remove excess *Agrobacterium*, and were placed into coculture medium top side down (CM) for 2 days in the dark at 28˚C. Finally, inoculated explants were transferred into regeneration medium (RM) (Table 1). The transgenic lines were transported to growth chamber at a photoperiod of 16:8 (L:D) hours at 25˚C, and then subcultured, each for 21 days.

**Table 1.** Composition of Culture Media ^a^

	GM	PM	CM	RM	IM
**MS**	+	+	+	+	+
**Sucrose, g/L**	30	30	30	30	50
**Acetosyringone, mM**			200		
**NAA, mg/L**			1		
**BAP, mg/L**		2	1		
**Zeatin Riboside, mg/L**				0.5	
**IAA, mg/L**				0.1	
**Cefotaxime, mg/L**				250	
**Hygromycin, mg/L**				7.5	

^a^ Abbreviations: Ms, Murashige and Skoog (1962); PM, preculture medium; NAA, 1-naphthaleneacetic acid; CM, coculture medium; BAP, 6-benzylaminopurine; RM, regeneration medium; IAA, indole acetic acid; IM, infection medium; GM: germination medium.

### 3.5. Genomic DNA Extraction and PCR Analysis of Transgenic Plants

Genomic DNA of transgenic plants was extracted, using the CTAB method (29). A quantity of 0.3 gram of fresh leaves of expected transgenic plant was used for the CTAB method and quality and quantity of purified genomic DNA was evaluated (30). PCR amplification of genomic DNA for presence of proinsulin gene was performed using the specific primers.

### 3.6. Dot Blot Analysis

For the extraction of total soluble protein (TSP), 200 mg of young tomato leaves were used. The tomato leaves were ground in liquid nitrogen to a fine powder. Proteins were extracted by using 1000 µL of extraction buffer (50 mM Tris-HCl, 2 mM Ethylene Diamine Tetra Acetic Acid (EDTA) and 0.04% (v/v) 2- Mercaptoethanol). Cell debris was removed by two rounds of centrifugation (24000 g, 21 minutes at 4˚C), and the supernatant was used for dot blot analysis. Twenty nanograms of protein samples were directly spotted onto a nitrocellulose membrane. BSA 1% solution was added after dying and incubated for 1 hour at 25˚C. Solution was poured out and washing with PBS-T for 5 minutes was performed; this step was repeated 3 times. Then the primary antibody (Santa Cruz Biotechnology, Inc.) was added and incubated for 1 hour. The solution was poured out; washing with PBS-T was performed for 5 minutes, and repeated 3 times. Next, nitrocellulose membrane was incubated in anti-rabbit IgG conjugated with horseradish peroxidase as a secondary antibody for an hour with 1:4000 dilutions. Color development solution was DAB and 0.01% hydrogen peroxide in 50 mM Tris (pH = 7.5).

## 4. Results

### 4.1. Gene Cloning and Construction of Recombinant Strain Containing pCAMBIA-Ins

**Figure 3.**
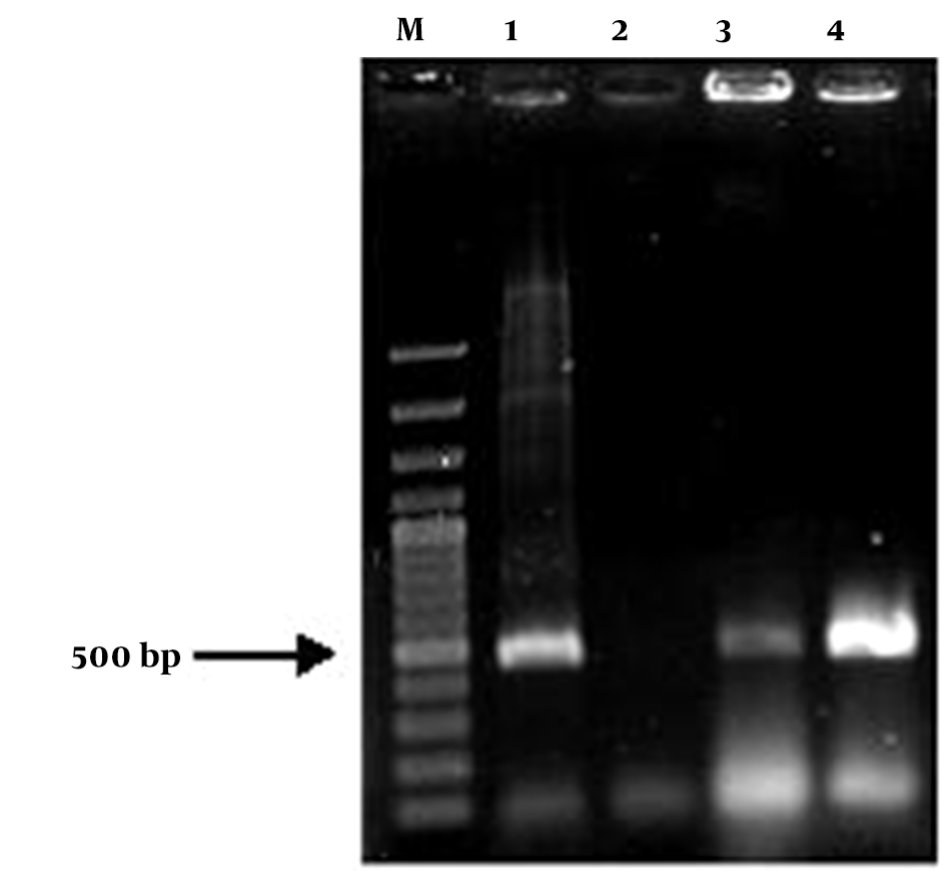
Affirmation of the Presence of Proinsulin Gene in pCAMBIA1304 by the Colony PCR Technique M, 100 bp marker; Lane 1, positive control; Lane 2, negative control; Lanes 3-4, random colonies selection.

**Figure 4.**
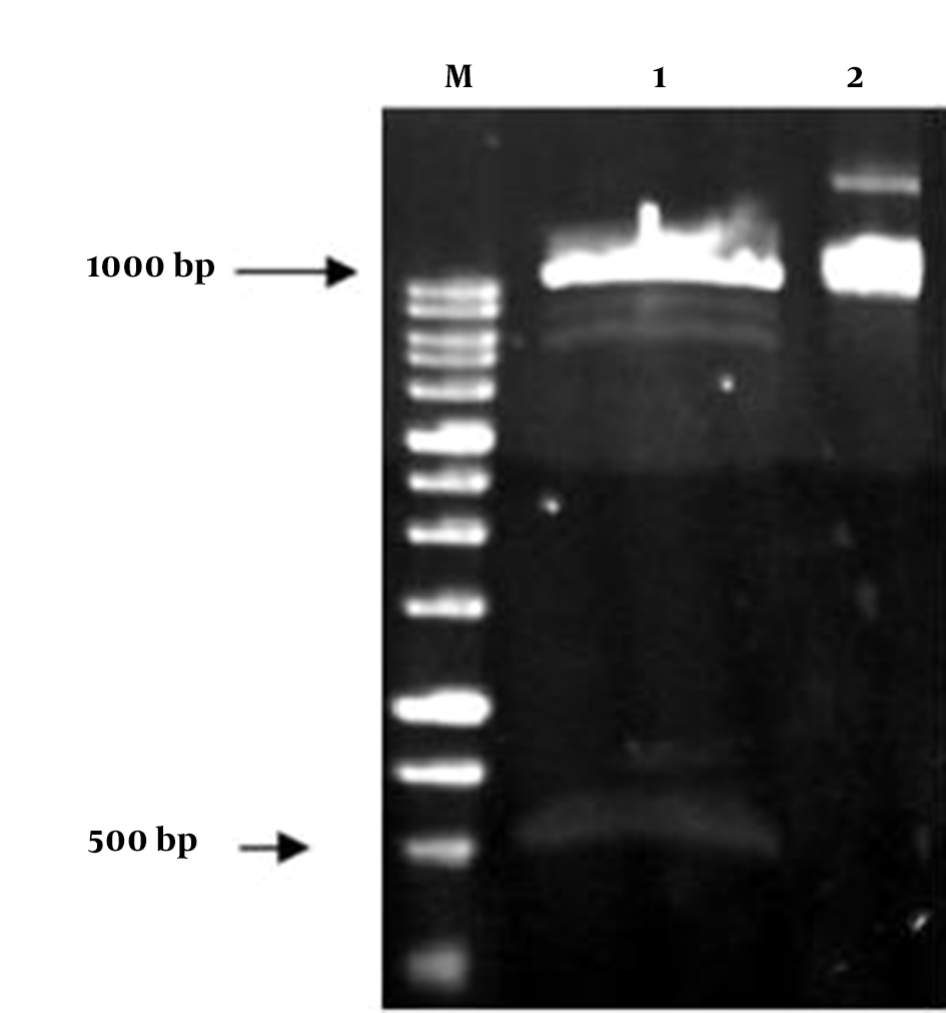
Affirmation of the Presence of Proinsulin Gene With Restriction Enzymes Digestion M, 1 kb marker; Lane 1, digestion of pCAMBIA1304 expression vector by Bst EII and Nco I restriction enzymes and proof of the presence of 500 bp band; Lane 2, the vector without digestion.

The insulin gene was cloned in plant binary vector pCAMBIA1304. The restriction sites NcoI and BstEII were added to the insulin gene sequence by designing primers containing these two restriction enzymes via the PCR technique. A 500-bp-long fragment of insulin PCR product was successfully cloned into the pCAMBIA1304 between CaMV35S promoter and NOS terminator, and in the positions of BstEII and NcoI restriction sites. Then, this construct was evaluated on agarose gel and transformed into *E. coli* strain DH5α. The constructed vector was confirmed by colony PCR, PCR, digestion and sequencing (Figures 3 and 4). The presence of the insulin gene was confirmed by colony PCR using the specific primers and showed a 500 bp band on agarose gel. The digestion reaction was performed by BstEII and NcoI restriction enzymes. The recombinant pCAMBIA-Ins construct was transformed into *Agrobacterium tumefaciens* strain LBA4404, and was confirmed by colony PCR and sequencing.

### 4.2. Plant Transformation

**Figure 5.**
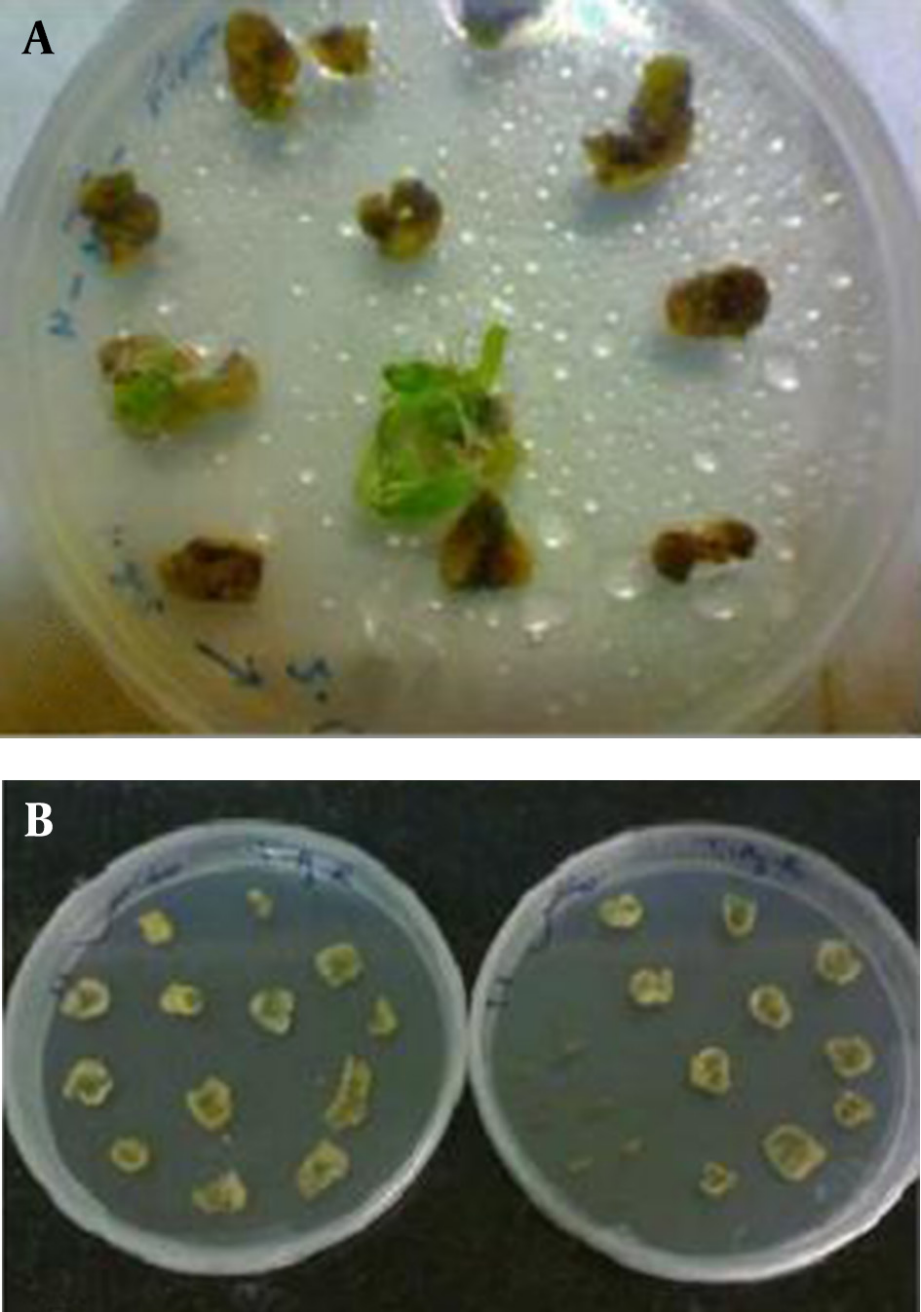
Leaves Were Inoculated by *Agrobacterium tumefaciens* A. with pCAMBIA-ins, on selection medium with hygromycin for selection of transformed plants after 3 weeks, and B. without pCAMBIA-ins as the control

**Figure 6.**
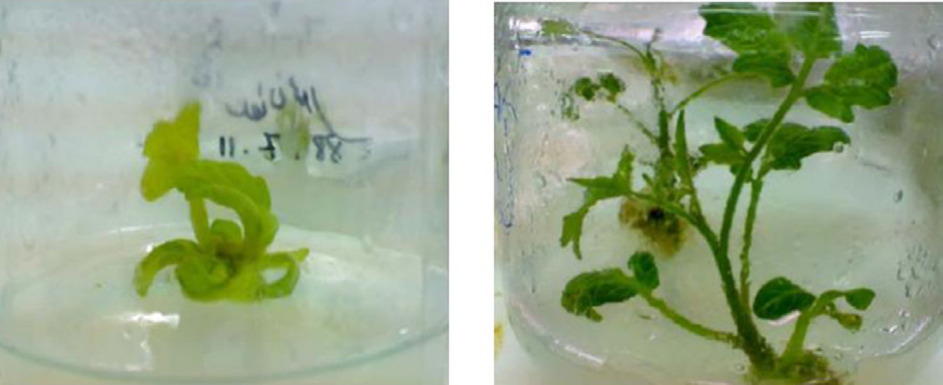
Transforming the Regenerated Plants Into the Tissue Culture Glass Bottles After 6 Weeks on MS Medium

After 2 to 3 times of subculturing, the cotyledonary inoculated explants showed regeneration, especially at the cutting edges, while control plants showed no regeneration (Figures 5 and 6).

### 4.3. PCR Analysis

**Figure 7.**
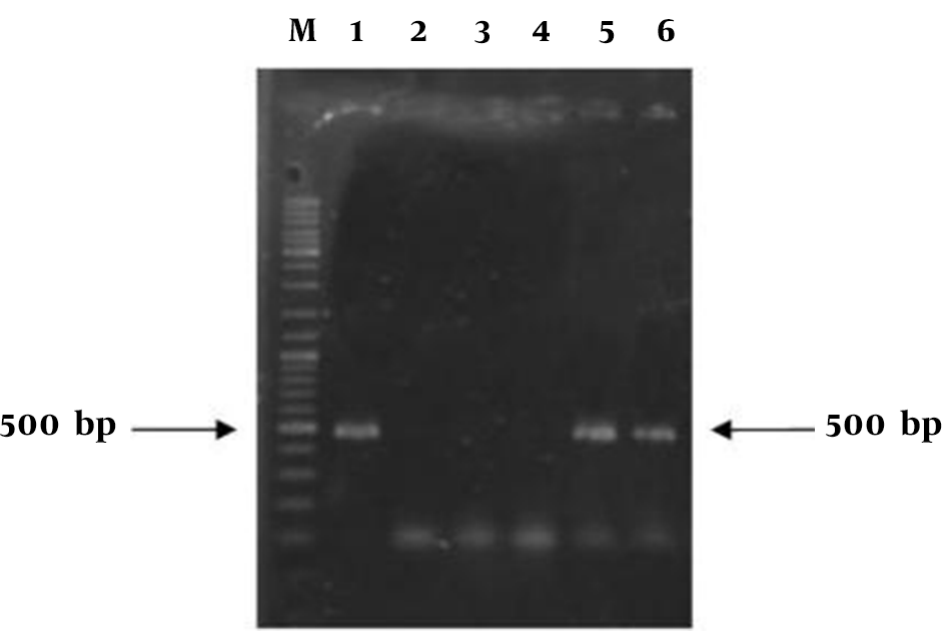
PCR Analysis of Expected Transgenic Plants M, 100 bp marker; Lane 1, positive control; Lane 2, ddH_2_O; Lane 3, wild type plant; Lane 4, no transgenic plant; Lanes 5 and 6, transgenic plants.

PCR based analysis with the primer pair was performed to examine the integration of the transgene into the genomic DNA. Presence of a 500 bp band in expected transgenic lines showed the successful integration of transgene into regenerated tomato plants (Figure 7). Untransformed plants (negative control) did not show any PCR product, which indicated that these plants have no transgene.

### 4.4. Analysis of Protein Expression by Dot Blotting

**Figure 8.**
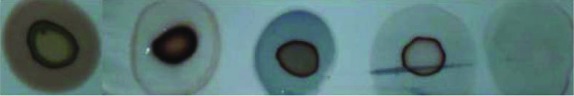
Dot Blot Analysis of Protein Extraction of Tomato 1. Positive control; 2, 3, 4. Protein extraction of transgenic plant; 5. Protein extraction of wild-type plant (negative control).

Selected transgenic plants by hygromycin resistance and PCR amplification were used for protein extraction from leaves for dot blot analysis. Development of brown color in each sample interpreted as a transgenic line. The difference between transgenic lines and wild-type plants confirmed the expression of proinsulin protein in transgenic lines. We used insulin protein as the positive control (Figure 8). 

## 5. Discussion

New studies have demonstrated that molecular farming in plants has many advantages regarding practical, economic and safety aspects. It is estimated that the use of plants can reduce the cost of production compared to other production systems like microbial fermentation systems (2-10% cost reduction) and mammalian cell cultures (0.1% cost reduction) (19). The first plant-made biologic approved by the FDA in 2012 was an enzyme (taliglucerase alfa) produced in genetically engineered carrot cells for treating type 1 Gaucher’s disease (31). The purpose of this study was cloning, transformation and expression of proinsulin in tomato. In this study, the proinsulin gene was cloned, transformed, and expressed in tomato. This vegetable is a competent system for producing many therapeutics like HBV, HIV and other recombinant proteins, because if these recombinant pharmaceutical proteins are expressed in fruits, they can potentially be used orally. This strategy can solve some plant expression system drawbacks like purification requirement and drug injection, etc. Tomato contains some other compounds like carotenoids, mineral salts, vitamins, amino acids, sugar and; hence it plays a key role in health. It has been shown that plant based pharmaceuticals have advantages compared to other conventional expression systems like microorganisms and cell culture. So the use of plant expression systems for large-scale biopharmaceutical production is becoming more accepted (12). Stable transformation is a better choice compared with transient expression since it generates transgenic lines which have the potential to produce millions of seeds (10). In this case plant expression vector pCAMBIA1304 was used which includes the CaMV35s promoter and NOS terminator transcription elements. Now the most popular promoter in dicotyledonous plants is CaMV35S. It is a powerful fundamental promoter which can be made even more active by duplicating the enhancer region (3). According to the importance of proinsulin and the benefits of tomato as a host plant for recombinant protein production, this expression platform can be suitable as an alternative system for production of human proinsulin protein.
